# Smartwatch-Based Tailored Gamification and User Modeling for Motivating Physical Exercise: Experimental Study With the Maximum Difference Scaling Segmentation Method

**DOI:** 10.2196/66793

**Published:** 2025-04-18

**Authors:** Jie Yao, Di Song, Tao Xiao, Jiali Zhao

**Affiliations:** 1 School of Economics and Management Harbin Institute of Technology (Shenzhen) Shenzhen China; 2 School of Mathematical Sciences Shenzhen University Shenzhen China

**Keywords:** tailored gamification, user segmentation, maximum difference scaling, MaxDiff, smartwatch, physical exercise

## Abstract

**Background:**

Smartwatch-based gamification holds great promise for enhancing fitness apps and promoting physical exercise; however, empirical evidence on its effectiveness remains inconclusive, partly due to “one-size-fits-all” design approaches that overlook individual differences. While the emerging research area of tailored gamification calls for more accurate user modeling and better customization of game elements, existing studies have relied primarily on rating scale–based measures and correlational analyses with methodological limitations.

**Objective:**

This study aimed to improve smartwatch-based gamification through an innovative user modeling approach to better motivate physical exercise among different user groups with tailored solutions. It incorporated both individual preferences and needs for game elements into the user segmentation process and used the maximum difference scaling (MaxDiff) technique, which can overcome the limitations of traditional methods.

**Methods:**

With data collected from 2 MaxDiff experiments involving 378 smartwatch users and latent class statistical models, the relative power of each of the 16 popular game elements was examined in terms of what users liked and what motivated them to exercise based on which distinct user segments were identified. Prediction models were also proposed for quickly classifying future users into the right segments to provide them with tailored gamification solutions on smartwatch fitness apps.

**Results:**

We identified 3 segments of smartwatch users based on their preferences for gamification. More importantly, we uncovered 4 segments motivated by goals, immersive experiences, rewards, or social comparison. Such user heterogeneity confirmed the susceptibility of the effects of gamification and indicated the necessity of accurately matching gamified solutions with user characteristics to better change health behaviors through different mechanisms for different targets. Important differences were also observed between the 2 sets of user segments (ie, those based on preferences for game elements vs those based on the motivational effects of the elements), indicating the gap between what people enjoy using on smartwatches and what can motivate them for physical exercise engagement.

**Conclusions:**

To our knowledge, this study is the first to investigate MaxDiff-based user segmentation for tailored gamification on smartwatches promoting physical exercise and contributes to a detailed understanding of preferences for, and the effectiveness of, different game elements among different groups of smartwatch users. As existing tailored gamification studies continue to explore ways of user modeling with mostly surveys and questionnaires, this study supported the adoption of MaxDiff experiments as an alternative method to better capture user heterogeneity in the health domain and inform the design of tailored solutions for more application types beyond smartphones.

## Introduction

### Background

The COVID-19 pandemic and its widespread consequences have brought public health issues and related preventive measures into sharper focus. Increasing efforts are being made to educate people on the harms of unhealthy lifestyles, which are closely associated with the leading global risks for mortality and chronic diseases such as high blood glucose and cholesterol levels [1,2]. One of the most prevalent health concerns in modern society is sedentary behavior, whereas adequate physical exercise can produce positive physical and mental effects [3,4]. Although many people are aware of such benefits, motivational techniques and systems are needed to push people to take action [5] and sustain long-term exercise engagement.

Gamification, a form of persuasive design [6] and a useful behavioral change support system [7], has gained increasing popularity in recent years [8] owing to its theoretical bases in motivation research (such as self-determination theory [9] and the behavior model proposed by Fogg [10]) and wide practical applications integrating motivational features such as goal setting and feedback. By incorporating common game design elements into nongame contexts to make activities more game like [11], gamification is believed to hold great promise in the area of health behavior change [12], particularly for promoting physical activity [13]. Despite the increasingly widespread use of gamification on many mobile fitness apps such as Nike+ Running (subsequently rebranded as Nike Run Club) and Zombies, Run! [14], the evaluation of such commercial apps and their metrics has been rare and inconclusive; for example, Direito et al [15] compared the use of Zombies, Run! with a nonimmersive app as well as the control group and did not find any significant intervention effect on the adolescent participants’ fitness and activity levels. In general, empirical evidence on the motivational effects of game elements has been accumulating and promising, although with heterogeneity across the relatively small number of high-quality experimental studies [16,17]. According to 2 most recent meta-analyses on gamification and physical activity [18,19], gamified interventions compared to a nongamified control promoted small increases in step count but not in more intense physical exercises; significant differences in the effect size were also observed among the reviewed studies, and it was suggested to provide tailored experiences for improving gamification effectiveness across diverse user populations. Overall, gamification in physical activity as a young area of research still faces many open questions that need to be rigorously studied, such as the effectiveness of various game elements, as well as the consideration of different application contexts and notable user differences [20].

### Tailored Gamification and User Modeling

One important reason for the discrepancies detected in existing gamification research is the reliance on the “one-size-fits-all” approaches adopted in many current apps. These approaches either did not consider variability across individuals due to user motivations and personal characteristics [21,22] or implemented too many game elements simultaneously [23], therefore yielding less effective results. Besides, the majority of prior studies focused on testing gamified systems with multiple elements together, providing limited information on the unique effects of individual elements [20,24].

To address these issues, there is an emerging area of interest called “tailored gamification,” which focuses on customizing game elements in given contexts to suit individual needs and preferences for the gamification experience [25]. Accordingly, a robust and accurate classification of users is a critical first step before any effective tailored gamification can be implemented. Meanwhile, current user modeling approaches rely primarily on rating scale–based measures as the basis for profiling, such as personality traits or player typologies [20], the latter being developed either in the context of games (eg, Bartle and BrainHex) or specifically for gamification (ie, Hexad) [26].

However, these popular measurement instruments possess methodological limitations for user segmentation and tailored gamification design. The most common issue inherent in rating scales is scale use bias, such as small ranges of mean item scores, a tendency for respondents to favor the top end of the scale, and scalar inequivalence across countries and cultures [27]. As rating scales do not require people to make trade-offs, they provide less accurate assessments of the relative importance of each item, making it harder to clearly differentiate one segment from another. A key example is the widely used gamification user types Hexad scale [28,29], which is a framework for categorizing individuals based on their motivations and preferences when interacting with gamified systems. The scale consists of 6 distinct user types: philanthropist, socializer, achiever, free spirit, player, and disruptor. Each is motivated by different aspects of gamification: purpose, relatedness, competence, autonomy, extrinsic rewards, and the triggering of change, respectively. Despite the helpfulness of the Hexad scale for understanding user diversity, the issues with rating scales are still unavoidable; for example, although users can be classified into 1 of the 6 types based on their highest scores, the score differences across the dimensions might not be conspicuous or practically meaningful, and there are also strong correlations between certain types, such as those between the philanthropist and socializer types [29]. Further evidence has shown that the Hexad user orientations are not necessarily stable and can change over time, suggesting that gamification design based on a 1-time measurement of the dominant Hexad user type might not be adequate [30].

In addition, while standard practices of correlation analysis can be used to match user types with their preferred game elements based on ratings [29], the accuracy of such methods is not guaranteed, given the limitations of rating scales again, as well as the challenge of determining the most powerful game elements from a large pool of suitable candidates for a given user type. As summarized by Klock et al [25], >20 different game elements were preferred by achiever, player, and socializer types, and 11 game elements were proposed even for the least common type, disruptor. More research is needed to tease out the unique effects of individual game elements to optimize their use for different user groups, particularly for tailored design on limited interfaces.

### Maximum Difference Scaling: An Alternative User Modeling Method

Given the aforementioned issues with existing user modeling approaches, alternative methods are needed to advance the field of tailored gamification. If the purpose of tailoring is to make gamification better fit what the users will enjoy or be motivated by, a natural way would be directly using user preferences and needs for various game elements as the basis for profiling. However, there is a lack of research using this intuitive approach, which might be due to the limitations of traditional rating and segmentation methods, such as scale use bias and statistical deficiencies [27]. Besides, the problem with asking users about game design elements directly, according to Tondello et al [29], is that users might not be aware of their game preferences and thus be unable to rate each element accurately. It is also important to examine psychological factors such as motivation in a gamified context beyond what users like, which can help determine what type of gamification works best for stimulating behavior change rather than merely pleasing the users.

Accordingly, the maximum difference scaling (MaxDiff) method, a widely used technique in marketing research, provides a useful alternative for classifying gamification users because it is “a rating method that does not experience scale use bias, forces trade-offs, and allows each scale point to be used once and only once” [27]. As a classic pairwise comparative analysis method [31], MaxDiff requires respondents to select 2 items from a given set—their most and least preferred—that can indicate the maximum difference in their preferences. This approach has been shown to solve the problem of scalar inequivalence across countries [32] and demonstrate greater discrimination among items and between respondents than Likert scales [33]. MaxDiff can also be conveniently integrated into scenario-based experiments that simulate real-world contexts and improve experimental results [34]. In addition, it generates valuable data for segmenting users into distinct groups.

The MaxDiff technique provides a great opportunity for tailored gamification research, which is still a new area at the very early stage of development, with the majority of research conducted in the education domain [35,36]. Limited evidence exists on the usefulness of MaxDiff for teasing out user preferences for game design elements [37,38]; however, its full potential for user segmentation remains underused. In general, user modeling for tailored gamification needs to “consider many characteristics simultaneously to evaluate how much one may influence the other” [25] to achieve better segmentation of gamification users. It should also consider the context of use that can affect the effectiveness of game elements (eg, task, domain, and device), with most existing research limited to education and smartphone apps [25]. More inquiry into the health domain and other new application types will be particularly valuable.

### Smartwatch-Based Gamification for Physical Activity

One promising health-related application of tailored gamification is its integration into popular wearable activity trackers such as smartwatches, which were predicted to be used by approximately 225 million people globally in 2024 [39] and hold great promise for promoting healthy lifestyles through useful functions such as health monitoring, reminders, and sharing [40]. In particular, a recent umbrella review confirmed that using wearable devices could significantly increase physical activity [41] and suggested leveraging motivational features to enhance the effectiveness of wearables. However, near half of the users abandoned their devices within the first 6 months [42,43]; yet, most related research has only focused on technical issues rather than user perceptions and preferences [44]. A small number of studies did point out the importance of providing tailored feedback and intrinsically motivating users [45], as well as optimizing design elements on the limited interfaces of smartwatches [46].

Meanwhile, gamification is believed to enhance personal informatics apps and sustain user engagement [47]. As an effective feedback mechanism [48], gamification can aid the interpretation of health monitoring data for users in a straightforward and motivating way. Moreover, unlike mostly self-reported data from mobile phone apps, smartwatches offer the distinct advantage of providing immediate feedback based on real-time objective health data. Their wearable nature also makes them more suitable for exercise contexts, thereby improving the effectiveness of gamification in a more accurate and timely fashion [16]; for example, gamification on smartwatches has been found effective for obesity control [49,50], while a systematic review of mobile health–based gamification interventions suggested the need for more empirical research to explore the efficacy of combining gamification and wearables for promoting physical activity in various populations [17]. In particular, tailored gamification has the potential to further improve engagement and motivation for exercising among all users [22].

As smartwatch users vary in their needs and behaviors, accurate user modeling is required to categorize them accurately and tailor different game elements for different groups to better motivate users for maintaining regular exercise engagement and lifelong healthy lifestyles. From a methodological perspective, the MaxDiff method is particularly useful for optimizing gamification design on the small smartwatch screen, which requires a nuanced understanding of trade-offs that different users make among various game elements (rather than the absolute importance of each element). Only on the basis of a clear understanding of these subtle elements and heterogeneous priorities can smartwatch users be accurately classified, who will then be offered tailored design solutions promoting both user enjoyment and health benefits through a carefully selected set of functions and displays on the limited interfaces of smartwatches.

### This Study

This study aimed to understand how smartwatch-based gamification should be tailored for different user groups to effectively promote physical exercise based on a more accurate and innovative user modeling approach. We incorporated both user preferences and needs for game elements from smartwatch users into the segmentation process and adopted the MaxDiff experimental method requiring users to make trade-offs among various game elements, followed by a quantified categorization of users into distinct groups using the latent class technique. As formulating theory-based hypotheses was not possible, given the limited prior research in the young area of tailored gamification, we proposed the following research questions (RQs):

- RQ1: What is the relative importance of each game element in smartwatch fitness apps based on the preferences of smartwatch users?
- RQ2: Which game elements in smartwatch fitness apps are more effective than others for motivating physical exercise among smartwatch users?
- RQ3: Are there distinct user groups with unique preferences for smartwatch-based gamification? If so, how can such segment membership be predicted among smartwatch users?
- RQ4: Are there distinct user groups who will be differentially motivated by smartwatch-based gamification? If so, how can such segment membership be predicted among smartwatch users?

## Methods

### Overview

As explained earlier, this study sought to compare the differences in user preferences and needs for gamification; therefore, using a simple sorting method was not appropriate due to the potential order effect in ranking that might bias the experimental results [27]. Meanwhile, if a Likert rating scale was used, respondents might assign similar preference scores to each game element, based on which the elements could not be clearly distinguished from one another. Therefore, we implemented the MaxDiff method and conducted 2 experiments to investigate user preferences for different game elements in smartwatch gamification apps. In addition, we compared the motivational effects of these elements on user willingness to exercise.

### Materials for the MaxDiff Experiments

**Figure 1:**
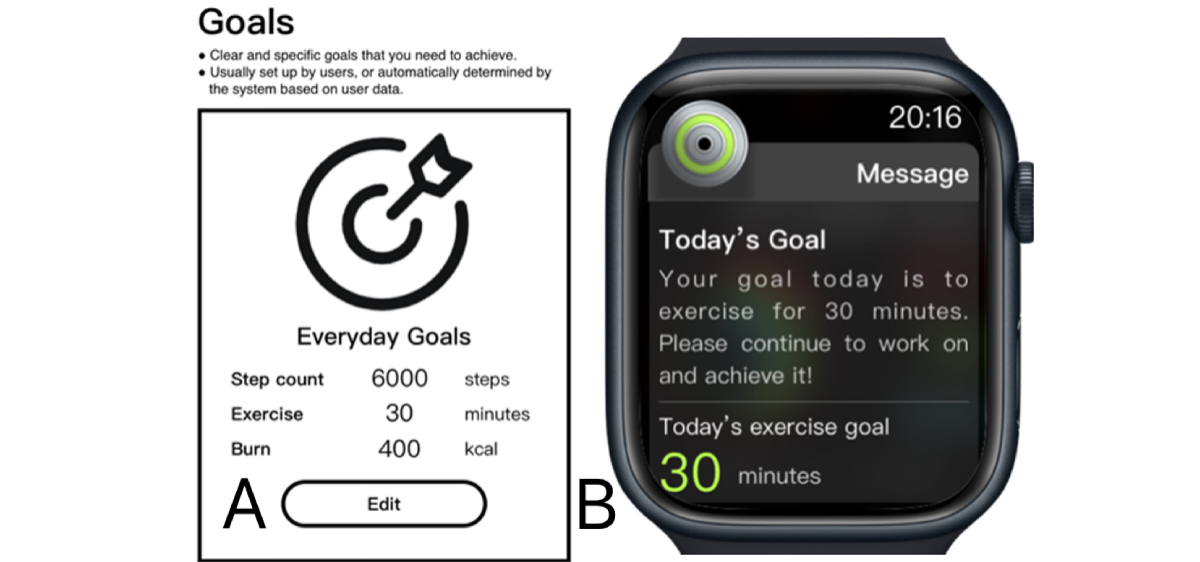
Samples of gamification materials for the MaxDiff experiments on (A) user preferences and (B) motivational effects.

On the basis of a comprehensive literature review and a preliminary study [51], we synthesized 16 commonly used game elements for the MaxDiff experiments: goals, progress, feedback, overview, points, levels, badges, leaderboards, community, sharing, cooperation, competition, challenges, narrative, avatars, and digital currency. These can be categorized into 4 groups: goal-related, reward-related, socialization-related, and immersion-related elements. After carefully reviewing related studies and market apps for the visualization and verbal explanation of each element, we designed 2 sets of experimental materials for the 2 experiments: a low-fidelity version for studying user preferences and a high-fidelity version for measuring the motivational effects of gamification (eg, the 2 versions of “goals” in Figure 1). The low-fidelity version adopted black-and-white wireframes to explain the concept of each game element in a simple manner to avoid the influence of color and design on user preferences. In comparison, the motivation-focused experiment was designed to simulate the real-life situation of using a smartwatch for exercising; therefore, the Apple Watch S6 prototype was used to represent the game elements in the form of various reminders displayed on a smartwatch screen. It should be noted that the text font and color were kept consistent across all materials for each game element to exclude the potential effects of such design factors on user responses.

### Other Instruments

In addition to conducting the MaxDiff experiments, we collected background data from users through survey questions as well as instruments for measuring gamification user types and motivation for smartwatch use (Multimedia Appendix 1). The Hexad scale [29] included 24 items to be rated on a 7-point scale ranging from 1 (*strongly disagree*) to 7 (*strongly agree*) to determine gamification user type, while the user motivation scale for smartwatch use [52] used a 6-point Likert scale and included 6 items, with 3 items measuring intrinsic motivation and 3 items measuring extrinsic motivation.

### Procedures

The study consisted of 3 parts: background information of users was collected first, followed by the preference-focused and motivation-focused MaxDiff experiments. Respondents were asked to provide basic information about themselves, including demographics, smartwatch use, exercise and gaming habits, and gamification user types. In particular, we collected important data about their attitudes and behaviors regarding smartwatch use, physical exercise, playing games, and gamification, which provided valuable complementary information for user segmentation.

The MaxDiff experiments started with a short sample MaxDiff exercise that helped familiarize the participants with the process so that they could quickly and accurately make their choices in the formal experiments that followed. In the first MaxDiff experiment, simple pictures and text descriptions about the 16 game elements related to a smartwatch fitness app (ie, the low-fidelity version) were presented to the participants based on an experimental design. Each time, participants were asked to repeatedly select their favorite and least favorite elements from the group of game elements presented. Specifically, the parameters of the MaxDiff experimental design were as follows: (1) 16 total items (game elements); (2) 4 items per question, following convention; (3) 12 questions per respondent to ensure that each item appeared at least 3 times; (4) 300 versions to meet the web-based questionnaire criteria; and (5) a minimum sample size of 200 to ensure that each item appeared at least 500 times. We followed the principle of balanced incomplete block design in selecting the MaxDiff experimental parameters, and the experimental design passed the merit criteria test, indicating that the design was reasonable and could be formally investigated.

**Figure 2:**
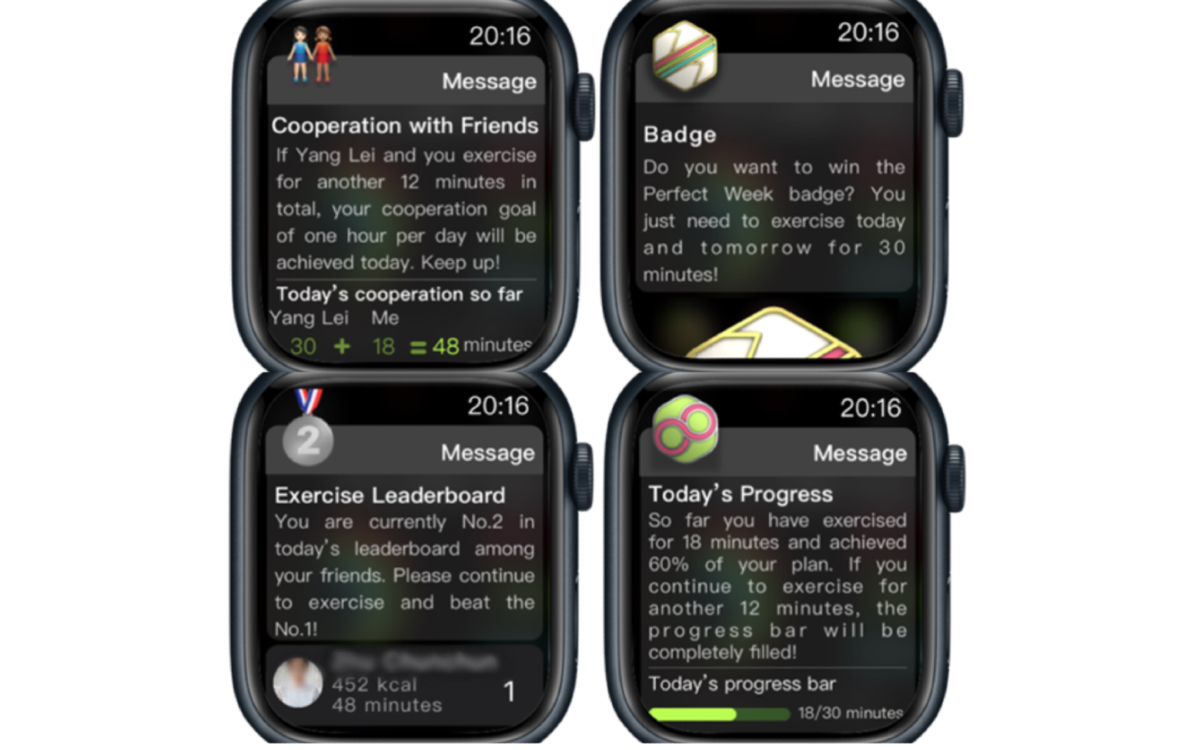
A sample scenario with 4 messages representing gamification elements from the motivation-focused MaxDiff experiment.

For the second, motivation-focused MaxDiff experiment, the parameters of the experimental design were exactly the same as those of the first experiment because the number of game elements selected were identical. The main difference lay in the contents and form of presentation: high-fidelity experimental materials were used, and the game elements were presented as context-specific reminders on a simulated smartwatch screen. We hypothesized the context for the participants as follows: “You are very tired after a long day of work and finally come home. You don’t want to exercise and just want to play with your smartphone, whereas a message alert suddenly pops up on your smartwatch.” In the 12 scenarios that followed, each composed of 4 messages representing gamification elements (refer to Figure 2 for a sample scenario), participants were asked to choose the messages that motivated them the most to start exercising and those that motivated them the least, which measured the motivational effects of different smartwatch-based game elements in the context of exercising.

### Sample and Data Collection

We used the specialized software Lighthouse Studio (Sawtooth Software) to design the MaxDiff experiments, program the measurement scales and survey questions, and collect data on the web. The study targeted smartwatch and smart band users aged 18 to 35 years in China. We recruited participants through invitations posted on popular social networking apps (eg, WeChat and QQ). A total of 529 people responded, of whom 378 (71.5%) passed the screener and provided valid responses. The whole study took 10 to 15 minutes to complete. Screening and data cleaning criteria included not meeting the background requirements (such as age not suitable and not having a smartwatch or smart band), incomplete responses, completion time <10 minutes, and straight-lining answers.

### Ethical Considerations

This study was approved by the ethics committee of Shenzhen University (2022010710). Informed consent was obtained electronically before participants began the web-based study, and participants could opt out at any time. All data collected were anonymous. Each participant received a cash incentive of CN ¥10 (US $1.5).

### Data Analysis

SPSS software (version 26.0; IBM Corp) was used for data cleaning and conventional statistical analysis, while data from the MaxDiff experiments were analyzed using Lighthouse Studio. Specifically, the utility scores for all users’ preferences for each game element were calculated using both logit analysis and hierarchical Bayes estimation. We also obtained the overall ranking of users’ preferences for the 16 game elements based on the rescaled preference scores which summed up to 100. Similarly, we analyzed the motivation-focused MaxDiff experiment data using the same approach, calculating the utility scores and rankings of the elements in terms of their motivational effects on users.

For the user segmentation, we performed latent class multinomial logit analysis on the MaxDiff data. As a powerful user segmentation method, latent class multinomial logit analysis is more suitable for analyzing MaxDiff data than other classification methods (eg, cluster analysis) because it is not solely data driven but based on statistical models. Users with similar choices were grouped into categories, and the part-worth utilities and the probability of each respondent belonging to each category were estimated for each category. After obtaining the preference-based and motivation-based segmentation schemes, we enhanced them by adding demographic information, smartwatch use, exercise and gaming habits, and gamification user types by means of chi-square tests and multivariate analysis of variance (MANOVA) to describe the specific characteristics of each user segment. Finally, significant factors that could help predict the future classification of users were also determined through multinomial logistic regression models.

## Results

### Descriptive Statistics

As shown in Table 1, our final sample consisted of 378 participants (male: n=204, 54%; female: n=174, 46%) with an average age of 23 (SD 2.916) years; 82 (21.7%) were employed, and the rest (n=296, 78.3%) were students. As shown in Table 2, of the 378 participants, 156 (41.3%) had prior experience using smartwatches, and 222 (58.7%) had prior experience using smart bands. The majority (293/378, 77.5%) had worn the device for >3 months with daily use. The main purpose of using the wearable varied from health monitoring and fitness to messaging and mobile assistance, with health monitoring and fitness reported by 224 (59.3%) of the 378 participants. Regarding physical exercise, 70.9% (268/378) of the participants did not meet the World Health Organization’s recommendation of exercising at least 3 times per week, with exercises limited to walking or aerobics. By contrast, the distributions of game playing preferences and behaviors were fairly even, suggesting notable individual differences in this area.

**Table 1:** Demographics of participants (n=378).

Characteristics		Participants, n (%)
**Age (y)**		
	18-22	154 (40.7)
	23-35	224 (59.3)
**Sex**		
	Male	204 (54)
	Female	174 (46)
**Occupation**		
	Student	296 (78.3)
	Employed	82 (21.7)

**Table 2:** Self-reported smartwatch use, exercise habits, and gaming behaviors of participants (n=378).

Variables		Participants, n (%)
**Device**		
	Smartwatch	156 (41.3)
	Smart band	222 (58.7)
**Duration of use**		
	<3 mo	85 (22.5)
	3-12 mo	128 (33.9)
	1-2 y	106 (28)
	≥3 y	59 (15.6)
**Frequency of use**		
	Wear occasionally	138 (36.5)
	Wear daily	240 (63.5)
**Main purpose of use**		
	Health monitoring	136 (36)
	Sports and fitness	88 (23.3)
	Messaging	68 (18)
	Mobile assistance	86 (22.8)
**Most frequently chosen exercises**		
	Walking	145 (38.4)
	Aerobics	135 (35.7)
	Other	98 (25.9)
**Exercise frequency**		
	≤3 times per mo	131 (34.7)
	1 to 2 times per wk	137 (36.2)
	≥3 times per wk	110 (29.1)
**Frequency of playing games**		
	Seldom	72 (19)
	Occasionally	133 (35.2)
	Frequently	99 (26.2)
	Daily	74 (19.6)
**Enjoyment of games**		
	Relatively dislike	219 (57.9)
	Relatively like	159 (42.1)

We also analyzed the scale measures. Table 3 presents the results for each Hexad user type and each of the 4 dimensions of gamification drive that we hypothesized, as well as motivation for smartwatch use. Among the Hexad user types, player, free spirit, and achiever had the highest average scores, followed by philanthropist and socializer, whereas disruptor had substantially lower average scores. Meanwhile, respondents reported higher scores in the goal-driven and achievement-driven dimensions in comparison with the immersion-driven and socialization-driven dimensions. Interestingly, intrinsic motivation for smartwatch use was higher than extrinsic motivation. It should be noted that all measures for each item in the scale were tested for normality for further statistical modeling.

**Table 3:** Scores for the Hexad user types, dimensions of gamification drive, and motivation for smartwatch use.

Items		Scores, mean (SD)
**Hexad user type**		
	Philanthropist	5.02 (0.915)
	Socializer	4.69 (1.071)
	Free spirit	5.48 (0.782)
	Achiever	5.26 (0.854)
	Disruptor	4.16 (1.031)
	Player	5.56 (0.901)
**Dimensions of gamification drive**		
	Goal driven	5.67 (0.930)
	Achievement driven	5.50 (0.975)
	Socialization driven	5.04 (1.159)
	Immersion driven	5.13 (1.153)
**Motivation for smartwatch use**		
	Intrinsic	4.68 (0.903)
	Extrinsic	3.98 (0.954)

### Overall MaxDiff Results

#### User Preferences for Gamification Elements

The results from the 2 analyses of the preference-focused MaxDiff data—logit analysis and hierarchical Bayes estimation—are compared in Table 4, showing overall consistency and negligible differences. It should be noted that we used the results of the logit analysis for further segmentation modeling to simplify the analyses and also because logit analysis is more commonly used in estimating mean utility scores and more applicable to studies involving a larger number of items [53].

**Table 4:** User preferences for each gamification element based on the preference-focused MaxDiff experiment.

Gamification element	Best (number of times)	Worst (number of times)	Logit analysis		Hierarchical Bayes estimation	
			Aggregate-level coefficient (rescaled)	Rank	Individual-level coefficient averaged (rescaled)	Rank
Goals	328	179	7.775	2	7.644	2
Progress	523	85	11.656	1	11.361	1
Feedback	263	220	6.561	7	6.205	7
Overview	338	244	7.126	4	7.188	3
Points	262	259	6.114	8	5.845	9
Levels	279	221	6.724	6	6.504	6
Badges	314	225	7.094	5	6.926	5
Leaderboards	268	301	5.748	10	5.874	8
Community	205	403	4.290	15	4.409	16
Sharing	251	275	5.848	9	5.506	11
Cooperation	254	289	5.716	11	5.766	10
Competition	234	353	4.959	12	5.162	13
Challenges	312	199	7.354	3	6.940	4
Narrative	271	450	4.432	14	5.244	12
Avatars	223	364	4.772	13	4.941	14
Digital currency	211	469	3.831	16	4.485	15

#### Motivational Effects of Gamification Elements

Similarly, based on the 2 aforementioned statistical approaches, we analyzed the average utility scores and rankings of the 16 elements for motivating users, as shown in Table 5. Interestingly, the order of importance among the elements differed from that in the first experiment, suggesting that user preferences did not necessarily equal the motivational power of gamification elements or predict actual behavior change; for example, it was surprising to see that cooperation as the 11th preferred element could indeed motivate users quite effectively, with a ranking of 4 among all 16 elements.

**Table 5:** Motivational effects of each gamification element based on the motivation-focused MaxDiff experiment.

Gamification elements	Best (number of times)	Worst (number of times)	Logit analysis		Hierarchical Bayes estimation	
			Aggregate-level coefficient (rescaled)	Rank	Individual-level coefficient averaged (rescaled)	Rank
Goals	389	120	9.181	2	8.876	2
Progress	541	71	11.985	1	11.663	1
Feedback	138	428	3.470	13	2.921	16
Overview	358	165	8.151	3	8.062	3
Points	289	214	6.738	7	6.521	9
Levels	283	211	6.721	8	6.583	8
Badges	324	207	7.275	5	7.188	5
Leaderboards	326	250	6.741	6	7.153	6
Community	142	474	3.177	15	3.005	15
Sharing	223	284	5.347	11	4.985	12
Cooperation	368	232	7.462	4	7.754	4
Competition	317	272	6.454	9	6.882	7
Challenges	247	220	6.233	10	5.762	10
Narrative	224	544	3.243	14	4.556	13
Avatars	140	494	3.049	16	3.073	14
Digital currency	227	350	4.773	12	5.015	11

### Segmentation Results Based on MaxDiff User Preferences

#### Model Estimation Results

To determine the fittest segmentation model with the best number of segments, multiple rounds of modeling were performed, with 7 metrics plus their log-likelihood values reported in Table 6 as measures of model fit. Specifically, users were classified into 2, 3, 4, and 5 categories according to their MaxDiff preferences for game elements, and the results were then compared based on a balanced evaluation of all 7 metrics in the following way. If relative chi-square was used as the fit-level criterion, 2 categories with the largest value should be selected as the classification result, whereas the optimal solution became 5 categories if percentage certainty and chi-square were used as the criteria. Similarly, if Akaike information criterion, consistent Akaike information criterion, Bayesian information criterion, and adjusted Bayesian information criterion were used as the criteria, 5 categories with the smallest value would be the best classification result. However, as 2 categories and 5 categories seemed to be extreme, we also found that the values of Akaike information criterion, consistent Akaike information criterion, Bayesian information criterion, and adjusted Bayesian information criterion decreased differently as the number of categories increased, with the greatest decrease occurring from 2 to 3 categories; therefore, it seemed most appropriate to classify users into 3 groups. In addition, the selection of the best number of categories should also consider other important factors, for example, whether the differences among the categories are obvious and whether the market sizes of each category are balanced. Therefore, based on the aforementioned criteria altogether, it was ultimately determined to best divide the users into 3 categories according to their distinct preferences for game elements, which was also supported by further analysis later.

**Table 6:** Model fitting tests for MaxDiff preference-based segmentation.

Metrics	Number of categories			
	2	3	4	5
Log-likelihood	–11,672.12	–11,338.98	–11,102.67	–10,930.04
Percentage certainty	7.19	9.84	11.72	13.09
AIC^a^	23,406.24	22,771.96	22,331.33	22,018.08
CAIC^b^	23,657.74	23,153.27	22,842.45	22,659.00
BIC^c^	23,626.74	23,106.27	22,779.45	22,580.00
ABIC^d^	23,528.23	22,956.91	22,579.24	22,328.95
Chi-square (*df*)	1808.7 (1)	2475.0 (2)	2947.6 (3)	3292.9 (4)
Relative chi-square (*df*)	58.3 (1)	52.7 (2)	46.8 (3)	41.7 (4)

^a^AIC: Akaike information criterion.

^b^CAIC: consistent Akaike information criterion.

^c^BIC: Bayesian information criterion.

^d^ABIC: adjusted Bayesian information criterion.

#### Preference Scores and Market Sizes by Segment

On the basis of the 3-segment model and the distinctive characteristics of each group, we categorized the 3 segments as goal-preferred, immersion-preferred, and reward-preferred segments. Table 7 shows by segment the market sizes and the logit preference coefficients for each gamification element, the latter being obtained from the latent class multiple logit analysis for each segment.

Clearly, the 3 segments differed significantly in their preference scores. Users in the goal-preferred segment scored notably higher on progress, goals, challenges, overview, and feedback than the other 2 segments but with much lower preferences for immersive elements such as avatars, narrative, and digital currency. In comparison, users in the immersion-preferred segment had much higher preference scores on narrative, avatars, badges, and challenges than those in the other 2 segments, who showed notably lower preferences for social elements such as community, sharing, competition, and leaderboards. By contrast, users in the reward-preferred segment loved reward-related elements and had the highest preference scores on digital currency, which was the least preferred by the other 2 segments. Not surprisingly, the reward-preferred users showed much lower preferences for social elements (eg, community, cooperation, and competition) as well as immersive elements (eg, avatars and narrative).

**Table 7:** Segment-level user preference coefficients for each gamification element and market sizes^a^.

Gamification elements	Goal-preferred segment	Immersion-preferred segment	Reward-preferred segment
Goals	9.529	6.322	6.175
Progress	13.977	9.324	9.806
Feedback	7.309	5.761	5.053
Overview	8.447	5.412	6.240
Points	4.058	4.283	11.426
Levels	6.982	5.470	6.572
Badges	7.307	6.998	5.825
Leaderboards	6.704	3.545	5.904
Community	2.887	5.026	4.654
Sharing	6.408	4.606	5.373
Cooperation	5.891	6.264	4.031
Competition	5.886	3.882	3.800
Challenges	9.471	6.355	4.703
Narrative	1.545	14.840	2.330
Avatars	2.243	9.823	4.123
Digital currency	1.356	2.090	13.986

^a^Market size (n=378): goal-preferred segment, n=161 (42.6%); immersion-preferred segment, n=113 (29.9%); and reward-preferred segment, n=104 (27.5%).

#### Demographic and Behavioral Characteristics of Each Segment

On the basis of the segmentation scheme, we also summarized the characteristics of each segment using descriptive statistics and chi-square tests, focusing on user demographics, physical exercise habits, and game playing. The goal-preferred segment had a higher proportion of male individuals than the other 2 segments, whereas the reward-preferred segment had a higher proportion of female individuals, although the sex differences were not statistically significant. Meanwhile, there was indeed a significant difference in age distribution among the 3 segments (*χ*^2^_2_=3.8; *P*=.049): of the 104 reward-preferred segment members, 72 (69.2%) were aged 23 to 35 years, a significantly higher proportion than in the other 2 segments. Occupations also differed significantly across the 3 segments (*χ*^2^_2_=7.6; *P*=.02), with a much smaller proportion of nonstudents (24/161, 14.9%) in the goal-preferred segment.

Furthermore, significant between-segment user differences were also observed regarding the most frequent exercise type (*χ*^2^_4_=12.1; *P*=.02), exercise frequency (*χ*^2^_4_=9.6; *P*=.048), and level of interest in exercising (*χ*^2^_4_=17.1; *P*=.002); for example, the goal-preferred segment had the smallest percentage of users (48/161, 29.8%) who identified walking as their most frequently chosen exercise, while the other 2 segments had higher percentages of users who preferred aerobic exercise. Meanwhile, the frequency of exercise was higher for the goal-preferred group and lower for the immersion-preferred group. Not surprisingly, there were more sports enthusiasts in the former segment but more users who hardly exercised in the latter segment. By contrast, no significant difference was detected among the segments in terms of liking games, although there was a higher percentage of game lovers in the immersion-preferred group.

In addition, we used 1-way MANOVA to analyze the differences among the segments in terms of Hexad user types and the 4 dimensions of gamification drive. First, we found that scores for the philanthropist (*P*<.001), socializer (*P*=.003), free spirit (*P*=.004), and achiever (*P*<.001) dimensions differed significantly across the segments, but there were no significant differences for the disruptor (*P*=.58) and player (*P*=.05) dimensions. As further detailed in Table 8, both the immersion-preferred and reward-preferred segments had significantly lower scores on the philanthropist dimension compared to the goal-preferred group, while on the socializer dimension, only the immersion-preferred segment scored significantly lower (*P*=.005). Similarly, on the free spirit and achiever dimensions, both the immersion-preferred and reward-preferred segments had significantly lower scores than the goal-preferred group.

**Table 8:** Multiple comparisons of the Hexad user type scores across the 3 preference-based user segments (where significant differences were detected; n=378).

Dependent variables, reference group, and segments			Participants, n (%)	Scores, mean (SD)	*P* value
**Philanthropist**					
	**Goal-preferred segment**				
		Goal preferred	161 (42.6)	5.23 (0.837)	—^a^
		Immersion preferred	113 (29.9)	4.94 (0.936)	.03
		Reward preferred	104 (27.5)	4.76 (0.940)	<.001
**Socializer**					
	**Goal-preferred segment**				
		Goal preferred	161 (42.6)	4.89 (1.031)	—
		Immersion preferred	113 (29.9)	4.45 (1.163)	.005
		Reward preferred	104 (27.5)	4.63 (0.974)	.11
**Free spirit**					
	**Goal-preferred segment**				
		Goal preferred	161 (42.6)	5.64 (0.645)	—
		Immersion preferred	113 (29.9)	5.39 (0.795)	.02
		Reward preferred	104 (27.5)	5.34 (0.918)	.02
**Achiever**					
	**Goal-preferred segment**				
		Goal preferred	161 (42.6)	5.56 (0.691)	—
		Immersion preferred	113 (29.9)	5.05 (0.855)	<.001
		Reward preferred	104 (27.5)	5.01 (0.936)	<.001

^a^Not applicable.

We also conducted multiple comparisons on the 4 dimensions of gamification drive, which showed that scores for the goal-driven (*P*<.001), achievement-driven (*P*<.001), socialization-driven (*P*=.02), and immersion-driven (*P*<.001) dimensions all differed significantly across segments. Specifically, as presented in Table 9, the immersion-preferred and reward-preferred segments had significantly lower scores than the goal-preferred segment on the goal-driven dimension. Not surprisingly, the goal-preferred and reward-preferred segments scored significantly lower on the immersion-driven dimension than the immersion-preferred group.

**Table 9:** Multiple comparisons of the dimensions of gamification drive across the 3 preference-based user segments (n=378).

Dependent variables, reference group, and segments			Participants, n (%)	Scores, mean (SD)	*P* value
**Goal driven**					
	**Goal-preferred segment**				
		Goal preferred	161 (42.6)	5.93 (0.778)	—^a^
		Immersion preferred	113 (29.9)	5.52 (0.892)	<.001
		Reward preferred	104 (27.5)	5.43 (1.084)	<.001
**Achievement driven**					
	**Goal-preferred segment**				
		Goal preferred	161 (42.6)	5.87 (0.826)	—
		Immersion preferred	113 (29.9)	5.26 (0.894)	<.001
		Reward preferred	104 (27.5)	5.19 (1.084)	<.001
**Socialization driven**					
	**Goal-preferred segment**				
		Goal preferred	161 (42.6)	5.23 (1.126)	—
		Immersion preferred	113 (29.9)	4.89 (1.118)	.04
		Reward preferred	104 (27.5)	4.91 (1.218)	.09
**Immersion driven**					
	**Immersion-preferred segment**				
		Goal preferred	161 (42.6)	4.89 (1.076)	<.001
		Immersion preferred	113 (29.9)	5.59 (1.110)	—
		Reward preferred	104 (27.5)	5.01 (1.179)	.001

^a^Not applicable.

#### Predicting Preference-Based User Segments by Logistic Regressions

To develop a useful method for quickly segmenting future users (ie, to assign them to a particular segment based on their answers to a few simple questions), we conducted rounds of multiple logistic regression modeling that included different combinations of variables from our survey. The final prediction model was composed of 2 dimensions of gamification drive (represented by achievement-driven and immersion-driven scores) and age group (divided into users aged 18-22 y and 23-35 y), with a good model fit (Table 10). Using the reward-preferred segment as the reference, age, achievement-driven scores, and immersion-driven scores could be significant predictors of particular user segments; for example, users aged between 18 and 22 years scoring high on the achievement-driven dimension or low on the immersion-driven dimension are more likely to belong to the goal-preferred segment rather than the reward-preferred segment. In comparison, users scoring high on the immersion-drive dimension are more likely to belong to the immersion-preferred segment.

**Table 10:** The final logistic regression models that predict preference-based user segments.

Segments^a^ and predictors			B		SE		*P* value		Exp(B)
**Goal-preferred segment**									
	Immersion driven scores	–0.283		0.126		.02		0.753	
	Achievement driven scores	0.915		0.158		<.001		2.497	
	Age (y): 18-22 (vs 23-35)	0.707		0.281		.01		2.027	
**Immersion-preferred segment**									
	Immersion driven scores	0.509		0.137		<.001		1.663	

^a^The reference category is the reward-preferred segment.

### Segmentation Results Based on MaxDiff Motivational Effects

#### Model Estimation Results

Similar to the segmentation process based on user preferences, we analyzed the 7 model-fit metrics with log-likelihood values when users were classified into 2, 3, 4, and 5 categories based on the MaxDiff results from the motivation-focused experiment (Table 11). Different models were compared, and we finally determined that dividing the users into 4 categories provided the best fit, following the same criteria and process as detailed in the previous subsection.

**Table 11:** Model fitting tests for MaxDiff motivation-based segmentation.

Metrics	Number of categories			
	2	3	4	5
Log-likelihood	–11,149.17	–10,803.67	–10,587.67	–10,432.31
Percentage certainty	11.35	14.10	15.81	17.05
AIC^a^	22,360.34	21,701.33	21,301.35	21,022.63
CAIC^b^	22,611.84	22,082.64	21,812.46	21,663.55
BIC^c^	22,580.84	22,035.64	21,749.46	21,584.55
ABIC^d^	22,482.32	21,886.28	21,549.26	21,333.50
Chi-square (*df*)	2854.6 (1)	3545.6 (2)	3977.6 (3)	4288.3 (4)
Relative chi-square (*df*)	92.1 (1)	75.4 (2)	63.1 (3)	54.3 (4)

^a^AIC: Akaike information criterion.

^b^CAIC: consistent Akaike information criterion.

^c^BIC: Bayesian information criterion.

^d^ABIC: adjusted Bayesian information criterion.

#### Motivation Scores and Market Size by Segment

On the basis of the 4-segment model and the distinctive characteristics of each group, we categorized the 4 segments as goal-motivated, immersion-motivated, reward-motivated, and socially motivated segments. Table 12 shows the market sizes of the 4 segments, together with the average utility scores of each game element’s motivational effect, which were calculated using logit scores, following the same approach as in the preference-based models.

The 4 segments also differed significantly in their motivation scores across the 16 game elements. Users in the goal-motivated segment were significantly more likely to be motivated by progress, goals, and overview than those in the other 3 segments. This segment, however, had much lower motivation scores on social gamification elements such as community, sharing, competition, and leaderboards. In comparison, users in the immersion-motivated segment scored significantly higher on narrative, badges, and avatars than the other 3 segments, suggesting that gameplay and fun elements were more effective in engaging them. Meanwhile, users in the reward-motivated segment were effectively motivated by reward-related elements such as digital currency, points, and levels, whereas immersive game elements such as avatars and narrative had less influence on them. It should be particularly noted that compared with the 3-segment scheme from the preference-based classification, a new segment was discovered in terms of the motivational effects of gamification, named the socially motivated segment. Users in this unique segment had significantly higher motivation scores than the other 3 segments on social elements such as leaderboards, competition, cooperation, and sharing but were the least influenced by immersive elements such as narrative and avatars.

**Table 12:** Segment-level motivational coefficients for each gamification element and market sizes^a^.

Gamification elements	Goal-motivated segment	Immersion-motivated segment	Reward-motivated segment	Socially motivated segment
Goals	13.981	6.214	6.983	10.171
Progress	15.228	9.572	9.754	13.171
Feedback	6.265	3.058	1.787	2.192
Overview	11.360	6.147	5.569	9.563
Points	7.241	5.809	12.106	3.630
Levels	9.009	5.670	7.141	4.886
Badges	9.969	8.069	5.163	4.611
Leaderboards	2.077	5.151	9.086	11.231
Community	1.354	4.007	4.408	1.687
Sharing	1.958	4.804	5.256	8.021
Cooperation	3.418	8.210	5.913	9.746
Competition	1.569	5.914	7.101	11.225
Challenges	9.384	4.423	4.363	6.699
Narrative	0.847	13.031	0.658	0.676
Avatars	2.510	5.225	1.605	1.132
Digital currency	3.830	4.695	13.108	1.359

^a^Market size (n=378): goal-motivated segment, n=79 (20.9%); immersion-motivated segment, n=128 (33.9); reward-motivated segment, n=64 (16.9%); and socially motivated segment, n=107 (28.3%).

#### Demographic and Behavioral Characteristics of Each Segment

On the basis of the segmentation scheme, we also summarized the characteristics of each segment using descriptive statistics and chi-square tests, focusing on user demographics, physical exercise habits, and game playing. The socially motivated segment had a higher proportion of male individuals (63/107, 58.9%) than the other 3 segments, whereas the reward-motivated segment had a higher proportion of female individuals, although the gender differences were not statistically significant. Meanwhile, there was indeed a significant difference in age distribution among the 4 user groups (*χ*^2^_3_=10.9; *P*=.01), with more users aged 23 to 35 years in the reward-motivated segment and more users aged 18 to 22 years in the socially motivated segment. The 4 segments also showed significant differences in the type of device used (*χ*^2^_3_=8.9; *P*=.03) and level of interest in exercising (*χ*^2^_6_=23.3; *P=*.001); for example, there were more smart band users in the socially motivated segment (75/107, 70.1%), which also featured more sports enthusiasts compared to many more users who hardly exercised in the immersion-motivated segment. Although there was no significant difference in terms of game playing, more users in the immersion-motivated segment preferred games compared to the other 3 groups.

In addition, we used 1-way MANOVA to analyze the differences among the segments in terms of Hexad user types and the 4 dimensions of gamification drive. First, we found that scores for the socializer (*P*=.001), free spirit (*P*=.02), and achiever (*P*<.001) dimensions differed significantly across the 4 segments, but there were no significant differences for the philanthropist (*P*=.08), disruptor (*P*=.40), and player (*P*=.12) dimensions. As further shown in Table 13, the goal-motivated and immersion-motivated segments had significantly lower scores than the socially motivated segment on both the socializer and achiever dimensions, while on the free spirit dimension, only the immersion-motivated segment scored significantly lower.

**Table 13:** Multiple comparisons of the Hexad user type scores across the 4 motivation-based user segments (where significant differences were detected; n=378).

Dependent variables, reference group, and segments				Participants, n (%)		Scores, mean (SD)		*P* value
**Socializer**								
	**Socially motivated segment**							
		Goal motivated	79 (20.9)		4.44 (0.954)		.007	
		Immersion motivated	128 (33.9)		4.53 (1.156)		.04	
		Reward motivated	64 (16.9)		4.92 (0.873)		.07	
		Socially motivated	107 (28.3)		4.93 (1.090)		—^a^	
**Free spirit**								
	**Socially motivated segment**							
		Goal motivated	79 (20.9)		5.60 (0.795)		.99	
		Immersion motivated	128 (33.9)		5.34 (0.788)		.02	
		Reward motivated	64 (16.9)		5.39 (0.870)		.40	
		Socially motivated	107 (28.3)		5.62 (0.677)		—	
**Achiever**								
	**Socially motivated segment**							
		Goal motivated	79 (20.9)		5.20 (0.914)		.01	
		Immersion motivated	128 (33.9)		5.03 (0.839)		<.001	
		Reward motivated	64 (16.9)		5.24 (0.917)		.06	
		Socially motivated	107 (28.3)		5.58 (0.682)		—	

^a^Not applicable.

We also conducted multiple comparisons on the 4 dimensions of gamification drive, which showed that scores for the goal-driven (*P*<.001), achievement-driven (*P*<.001), socialization-driven (*P*=.009), and immersion-driven (*P*<.001) dimensions all differed significantly across the 4 segments. Specifically, as presented in Table 14, the goal-motivated and socially motivated segments had significantly higher scores than the immersion-motivated segment on both the goal-driven and achievement-driven dimensions. On the socialization-driven dimension, only the socially motivated group scored significantly higher than the immersion-motivated segment. However, the immersion-motivated segment had a much higher score on the immersion-driven dimension than almost all other segments, with strong statistical significance, except for a borderline significance (*P*=.05) compared to the reward-motivated segment.

**Table 14:** Multiple comparisons of the dimensions of gamification drive across the 4 motivation-based user segments (n=378).

Dependent variables, reference group, and segments				Participants, n (%)		Scores, mean (SD)		*P* value
**Goal-driven dimension**								
	**Immersion-motivated segment**							
		Goal motivated	79 (20.9)		5.79 (0.949)		.03	
		Immersion motivated	128 (33.9)		5.41 (0.938)		—^a^	
		Reward motivated	64 (16.9)		5.68 (1.021)		.39	
		Socially motivated	107 (28.3)		5.89 (0.774)		<.001	
**Achievement-driven dimension**								
	**Immersion-motivated segment**							
		Goal motivated	79 (20.9)		5.58 (1.020)		.03	
		Immersion motivated	128 (33.9)		5.18 (0.980)		—	
		Reward motivated	64 (16.9)		5.46 (0.989)		.33	
		Socially motivated	107 (28.3)		5.85 (0.796)		<.001	
**Socialization-driven dimension**								
		**Immersion-motivated segment**						
		Goal motivated	79 (20.9)		4.90 (1.197)		.99	
		Immersion motivated	128 (33.9)		4.85 (1.196)		—	
		Reward motivated	64 (16.9)		5.11 (1.121)		.59	
		Socially motivated	107 (28.3)		5.33 (1.055)		.008	
**Immersion-driven dimension**								
	**Immersion-motivated segment**							
		Goal motivated	79 (20.9)		4.92 (1.321)		.009	
		Immersion motivated	128 (33.9)		5.49 (1.101)		—	
		Reward motivated	64 (16.9)		5.05 (1.101)		.05	
		Socially motivated	107 (28.3)		4.91 (1.012)		<.001	

^a^Not applicable.

#### Predicting Motivation-Based User Segments by Logistic Regressions

With the same aim of quickly assigning future users to a particular motivation-based segment using a few simple questions, we again conducted rounds of multiple logistic regression modeling that included different combinations of variables from our survey. The final prediction model was composed of scores from 3 Hexad dimensions (ie, socializer, free spirit, and achiever), the score on the immersion-driven question, age group (divided into users aged 18-22 y and 23-35 y), and level of interest in exercising (1=hardly any exercise, 2=general sports crowd, and 3=sports enthusiasts), with a good model fit (Table 15). Using the reward-motivated segment as the reference group, the aforementioned variables could be significant predictors of particular user segments; for example, users aged between 18 and 22 years scoring high on the achiever dimension are more likely to belong to the socially motivated segment rather than the reward-motivated segment. In comparison, users scoring high on the immersion-driven dimension or having at least some interest in exercising are more likely to belong to the immersion-motivated segment.

**Table 15:** The final logistic regression models that predict motivation-based user segments.

Segments^a^ and predictors			B		SE		*P* value		Exp(B)
**Socially motivated segment**									
	Achiever	0.600		0.260		.02		1.822	
	Age (y): 18-22 (vs 23-35)	1.185		0.354		.001		3.272	
**Goal-motivated segment**									
	Free spirit	0.841		0.290		.004		2.318	
	Socializer	–0.613		0.196		.002		0.542	
**Immersion-motivated segment**									
	Socializer	–0.367		0.183		.04		0.693	
	Immersion driven scores	0.468		0.163		.004		1.596	
	General sports crowd	1.103		0.555		.047		3.013	
	Sports enthusiasts	0.922		0.428		.03		2.515	

^a^The reference category is reward-motivated segment.

### Comparison of MaxDiff Results Among the Hexad Types

In addition to the 2 sets of MaxDiff-based segmentation results, we also examined the 6 Hexad user groups in terms of their gamification preferences as well as motivators for behavioral change among the 16 elements, given the popularity of the Hexad scale in prior research for assessing gamification user types. Compared to the MaxDiff-based segments, the Hexad types were less clearly differentiated in both user preferences and the motivational power of game elements; for example, “progress” always appeared as the top-selected element, with similar scores across all 6 Hexad types (Table 16), whereas the immersion-preferred or reward-preferred segments exhibited much stronger preferences for other elements (Table 7). These results highlight the limitations of using Hexad types alone as the basis for user modeling and tailored gamification, suggesting that the MaxDiff method may be more effective in teasing out the relative importance of gamification elements and accurately matching them with the right users.

**Table 16:** User preferences for, and the motivational effects of, the gamification element “progress” among the 6 Hexad types (n=378).

Hexad type	Participants, n (%)	Hierarchical Bayes estimation scores, mean (SD)	
		Preference	Motivation
Philanthropist	43 (11.4)	11.49	12.06
Socializer	91 (24.1)	11.28	11.37
Free spirit	33 (8.7)	11.63	12.78
Achiever	47 (12.4)	12.00	11.78
Disruptor	113 (29.9)	11.07	11.29
Player	51 (13.5)	11.30	11.83

## Discussion

### Principal Findings

On the basis of 2 MaxDiff experiments and the latent class statistical technique, this study contributes to the growing body of research on user modeling for tailored gamification. Specifically, the relative power of each of the 16 popular game elements was examined in terms of what users preferred to see on smartwatch-based fitness apps as well as what motivated them to exercise, with interesting differences observed between the 2 approaches. More importantly, these factors formed the basis for identifying and predicting user groups with distinct characteristics as future targets of tailored gamification on wearables.

When smartwatch users were making trade-offs among different game elements based on their preferences (ie, RQ1), we found that progress, goals, challenges, overview, and badges were the top 5 favored elements, whereas digital currency, community, narrative, avatars, and competition were the least preferred. This ranking suggests the overall importance of goal setting and constant feedback for attracting users to gamification, who seem to care less about immersive features such as narrative and avatars commonly found in digital games. These overall preferences were also mostly consistent with the ranking of game elements as motivators for exercising (ie, RQ2), where immersion-related gamification was still less effective, while progress, goals, overview, and badges remained in the top 5. However, cooperation rose to be a powerful motivator (ie, ranked fourth) despite being less preferred by the users (ie, ranked 11th), indicating the potential of social gamification for stimulating behavioral change regardless of user preference. Similar to the contradictory results on the gap between the experiences of enjoyment and the motivational benefits of social gamification on an educational platform [54], this finding suggested that the theoretically presumed relationship between gamification preferences and motivators might not hold in the health context either.

While it is already useful to quantify the overall importance of each individual game element, more valuable findings arose from the MaxDiff-based segmentation models, showing huge heterogeneity among the smartwatch users in terms of both user preferences (ie, RQ3) and the motivational effects of gamification (ie, RQ4), which have confirmed the problem of the one-size-fits-all gamification approach found in many prior studies [22]. Each user segment displayed distinct personal characteristics as well as preferences and needs for gamification; for example, although the overall rankings of immersive game elements were low, the immersion-preferred segment scored particularly high on narrative as well as avatars, 2 main features for engaging players in digital games. This segment also differed significantly from the goal-preferred segment in user characteristics and behaviors, with a much lower frequency of exercise, a much lower proportion of sports enthusiasts, and much higher scores on the immersion-driven scale, suggesting the need for developing more immersive gamification solutions on smartwatches to attract this group. Furthermore, the motivation-based segmentation results showed narrative as the key element prompting the immersion-motivated segment to exercise, whereas the other 3 segments unanimously ranked this element as the least effective, with extremely low scores. Such notable differences further supported the necessity of tailored gamification based on accurate user modeling [25] to motivate health behavior change through different mechanisms for different targets.

In addition, important differences were also observed between the 2 sets of user segmentation results (ie, 3 preference-based segments vs 4 motivation-based segments). Although users in general displayed a weak preference for socialization-related elements and did not form a socialization-preferred group, such elements did play a key role in prompting the socially motivated user segment to exercise, which suggested the importance of tailored design with elements such as competition and leaderboards for those who do not necessarily like social comparison but can be motivated by such pressure. In comparison, goals and progress were ranked the highest by both the goal-preferred and goal-motivated segments, while the reward-preferred and reward-motivated segments were influenced the most by digital currency and points. Interestingly, although digital currency was the least preferred element by the goal-preferred and immersion-preferred segments, it still exerted some motivating influences on the goal-motivated and immersion-motivated segments (ie, at least not among the bottom 5 elements). Such discrepancies further indicate the gap between what people like to use and what motivates them subconsciously, which contributes to the research needed on understanding “whether the motivationability and benefits from gamification coincide with experiences of enjoyment as is theoretically presumed” [54] and can provide valuable guidance for developing apps aimed at changing behaviors rather than merely entertaining users.

### Implications for Research and Practice

As the majority of prior studies focused on gamified systems with multiple game elements combined, and the scope of elements examined was limited [20], this study applied an innovative approach to quantify the individual effects of major game elements in the context of smartwatch-based fitness apps, which can advance our understanding of what might or might not work in health gamification; for example, for the widely adopted trinity of gamification design elements—points, badges, and leaderboards [55]—only badges appeared in the top 5 elements in terms of either user preference or motivational influence. In comparison, progress and goals were overall more favored and effective, supporting the importance of goal setting and feedback in health gamification [8]. In addition, smartwatch users overall seemed to care the least about common components of digital games—such as narrative, avatars, and digital currency—which have been inadequately examined in existing gamification research [16].

However, this was only part of the picture. The overall rankings of game elements did not hold across different types of users who indeed displayed quite distinct preferences and needs for gamification. As such, the key contribution of this study is to the emerging research area of tailored gamification [25], which hinges on addressing the problem of “one-size-fits-all” gamification and capturing the types of individual differences to improve health outcomes such as physical activity. Prior systematic reviews [18,19] have pointed out the small, positive effect of gamification on step count but not on moderate to vigorous physical activity, with limited existing research on the latter. This study, given the findings from the second motivation-based MaxDiff experiment that measured users’ tendency to exercise, provided new evidence on the potential of gamification to effectively promote physical exercise beyond steps, particularly so with tailored solutions for different user segments. As gamification in physical activity is still a young area of inquiry, more field research should target the challenging but important outcome of regular physical exercise and leverage the principles of personalization and tailoring [56,57] for enhancing intervention effectiveness across diverse user populations. Regarding user modeling, researchers have suggested that incorporating more precise and relevant information, such as motivational and behavioral factors, to determine user types will help better explain heterogeneity among users and interventions [58,59], while this study clearly demonstrated the power of MaxDiff-based segmentation for tailoring smartwatch-based gamification.

In addition, both experiential and instrumental outcomes are needed in the context of health gamification design [60], that is, users should not only like the gamification experience but also be motivated to engage in a target activity because motivation is a major factor for health behavior change. Taking both user preferences and motivations into account, this study proposed a new categorization of smartwatch users in relation to gamification design elements on wearable fitness apps and developed prediction models for easily classifying future users into the right segments and accordingly designing more accurate, targeted solutions.

From a methodological perspective, our research also supported the usefulness of MaxDiff experiments for teasing out the relative importance of each individual game element [37,38] and confirmed the feasibility of MaxDiff-based user segmentation in future studies on tailored gamification. The MaxDiff method has been widely used in marketing research and proven advantageous over traditional rating scale questions [61,62], yielding more accurate responses. Moreover, such responses can be easily integrated into user segmentation models (such as latent class models) and contribute to higher classification efficiency. Therefore, MaxDiff experiments hold great promise in helping researchers quantify individual differences more efficiently and informing designers on the development of optimized solutions for different users. Specifically, this study demonstrated that MaxDiff segmentation achieved better differentiation than popular scales, such as Hexad [29], for assessing gamification user types, offering an alternative user modeling approach for better tailored gamification. An additional benefit of the MaxDiff method for future studies is to enable further analysis using the total unduplicated reach and frequency technique [53] to obtain an optimized set of game elements that simultaneously considers preferences from all user segments and maximizes the utilities for all users. This can help alleviate the problem of one-size-fits-all approaches when tailored solutions are not readily available.

### Limitations

MaxDiff experiments and the latent class segmentation technique for classifying smartwatch users are both novel and promising, but the results of this study should be interpreted in the context of a few limitations. With the burgeoning research attention devoted to gamification in recent years, new innovative game elements might be quickly developed and implemented on a wide range of apps on various platforms, making it challenging to come up with an exhaustive list of all elements for the quantification of individual effects. There are also limitations regarding the maximum number of attributes allowed to be tested in MaxDiff experiments. Therefore, based on a comprehensive literature review and a preliminary study [51], we tried our best to synthesize a total of 16 popular and important game elements, which hopefully could represent current mainstream trends in gamification research and practice and were particularly applicable to smartwatch-based fitness apps. If needed, future studies can continue to investigate novel game elements not considered in this research that might rise in popularity and deserve more attention.

Another limitation is that, given the exploratory nature of this study and to achieve a straightforward categorization of smartwatch users, our user segmentation modeling scheme did not include user characteristics beyond their preferences and needs for gamification. While more complex user models can be developed in the future, we did evaluate each user segment in terms of related attitudes and behaviors, which added details to the characteristics of each segment and also helped us determine key variables for predicting segment membership. This information provides the basis for the next key step in tailored gamification, that is, to transform the theoretical understanding of individual differences to apps that can detect them efficiently and provide tailored solutions accordingly in real practice.

### Conclusions

Despite the increasingly widespread use of gamification in digital health apps, empirical evidence on its effectiveness remains promising but inconclusive due to a lack of methodological rigor and the insufficient consideration of individual differences in many studies. To our knowledge, this study is the first to investigate MaxDiff-based user segmentation for tailored gamification on smartwatches promoting physical exercise—one of the most frequently encouraged yet inadequately adopted health behaviors worldwide. By quantifying the unique importance of each individual game element and improving the accuracy of user segmentation, our research identified 3 segments of smartwatch users based on their preferences for gamification in fitness apps. More importantly, we uncovered 4 segments motivated by goals, immersive experiences, rewards, or social comparison. Such user heterogeneity confirmed the susceptibility of the effects of gamification [8] and informed researchers and practitioners of the need to match gamified solutions with user characteristics for ensuring the effectiveness of gamification. As existing tailored gamification studies continue to explore ways of user modeling with mostly surveys and questionnaires [25], this study supported the adoption of MaxDiff experiments as an alternative method and contributed to the methodological rigor of this emerging area of inquiry, particularly in the health domain and for more application types beyond smartphones.

## Data Availability

The datasets generated and analyzed during this study are available from the corresponding author on reasonable request.

## Appendix

Multimedia Appendix 1 Smartwatch-based gamification questionnaire.

## Abbreviations

MANOVA: multivariate analysis of variance
MaxDiff: maximum difference scaling
RQ: research question
