# Supplementary material for: Smartwatch-Based Tailored Gamification and User Modeling for Motivating Physical Exercise: Experimental Study With the Maximum Difference Scaling Segmentation Method
Source: JMIR Serious Games. 2025 Apr 18;13:e66793. doi: 10.2196/66793 (PMC12048785; doi:10.2196/66793)
Supplement: Multimedia Appendix 1 [file games_v13i1e66793_app1.docx]

**Smartwatch-Based Gamification Questionnaire**

(Note: Translated from the Original Questionnaire in Chinese)

Dear Sir/Madam,

Hello! We are conducting a study on the "Gamification Design of Smartwatch Fitness and Health Apps," and we appreciate your participation in this survey experiment. The survey is anonymous, and the valuable feedback and information you provide will only be used for academic research purposes. Your information will remain confidential.

The survey should take about 15 minutes to complete and will be conducted online. We sincerely thank you for your support and cooperation. After completing the survey, you will receive a reward of 10 RMB!

**Section 1: Screener**

1. Have you ever used a smartwatch/smart band?
   A. Yes
   B. No (End the survey and thank you)

**Section 2: Device Usage**

1. The smartwatch/smart band you are currently using (or have used) is:
   A. Apple Watch
   B. Xiaomi Watch/Redmi Watch
   C. Huawei Watch
   D. Oppo Watch
   E. Samsung Galaxy Watch
   F. Xiaomi Band
   G. Huawei Band
   H. Other (Please specify)
2. How long have you been using a smartwatch/smart band?
   A. <3 months
   B. 3-12 months
   C. 1-2 years
   D. >=3 years
3. How often do you use a smartwatch/smart band?
   A. Never wear it
   B. Only wear it during exercise
   C. Wear it occasionally
   D. Wear it daily
4. What is the main purpose for using a smartwatch/smart band?
   A. Sports and fitness
   B. Health monitoring
   C. Message
   D. Mobile assistance
   E. Other (Please specify)
5. (Multiple choices) What features do you commonly use on your smartwatch/smart band?
   A. Fitness tracking
   B. Message notifications
   C. Heart rate monitoring
   D. Step tracking
   E. Sleep tracking
   F. Blood oxygen monitoring
   G. Calorie tracking
   H. Sedentary reminders
   I. Menstrual cycle tracking
   J. Payment
   K. Reminder alerts
   L. Alarm clock
   M. Weather
   N. Other (Please specify)

**Section 3: MaxDiff Exercise**

Suppose your smartwatch/smart band has a fitness and health application, we will now introduce several possible gamification features to understand your preferences.

Of the four features shown on this page, which one do you like the most, and which one do you like the least? (relatively)

(Four sets of such features in total)

**Section 4: MaxDiff User Preferences**

Thank you for your patience. We hope the previous exercise has given you a preliminary understanding of various gamification features.

Next, we would like to conduct a small experiment to further understand your preferences. There are twelve questions, and please answer them honestly. Thank you.

1. Suppose you are using a fitness and health application on a smartwatch, which of the four gamification features on this page would you like the most and which one would you like the least? (relatively)
   (12 questions in total, according to the MaxDiff experimental design)
2. (Multiple choices) Suppose your smartwatch/smart band has a fitness and health application, which gamification features would you like it to include?
   A. Community
   B. Levels
   C. Goals
   D. Cooperation
   E. Virtual currency
   F. Points
   G. Competition
   H. Virtual characters
   I. Challenge
   J. Leaderboards
   K. Overview
   L. Feedback
   M. Sharing
   N. Progress
   O. Badges
   P. Narrative

**Section 5: Exercise Habits**

Thank you for completing the previous experiment. We would now like to briefly understand your current exercise habits and the use of your smartwatch/smart band.

1. What is your main exercise goal?
   A. Maintain health
   B. Weight loss
   C. Build muscle
   D. Relaxation and stress relief
   E. Hobby
   F. Other (Please specify)
2. What is your most frequently chosen exercises?
   A. Take a walk
   B. Running
   C. Jump rope
   D. Swimming
   E. Hiking
   F. Cycling
   G. Strength training (machines, bodyweight exercises, etc.)
   H. Flexibility training (dance, yoga, aerobics, etc.)
   I. Team sports (basketball, football, volleyball, etc.)
   J. Racquet sports (table tennis, badminton, tennis, etc.)
   K. Other (Please specify)
3. What is your current exercise frequency?
   A. 1 time per month or less
   B. 2-3 times per month
   C. 1-2 times per week
   D. 3-5 times per week
   E. About once per day
4. How would you describe yourself in terms of exercise?
   A. Almost no exercise
   B. Casual exerciser (exercises occasionally)
   C. Fitness enthusiast (enjoys exercising)
   D. Exercise fanatic
5. Please rate your agreement with the following statements based on your experience using a smartwatch/smart band.
   (Scale from 1: Strongly disagree to 6: Strongly agree)

| Statement | Rating |
| --- | --- |
| I use my activity tracker because I want to learn more about my physical activity. | 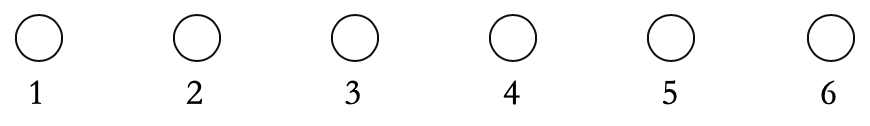 |
| I use my activity tracker because reaching my step or activity goals encourages me. | 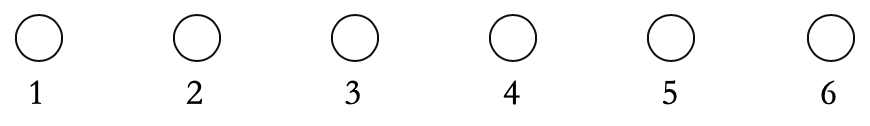 |
| I use my activity tracker because I find it interesting to deal with my activity data. | 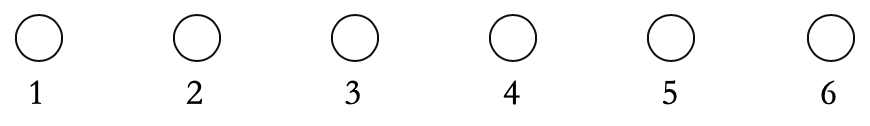 |
| I use my activity tracker because it is fun to deal with my activity data. | 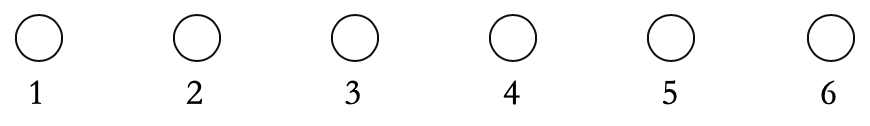 |
| I use my activity tracker to avoid taking too little exercise. | 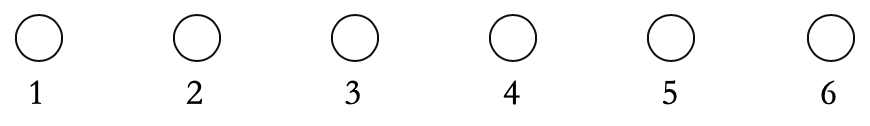 |
| I use my activity tracker because it assists me in taking care of my physical fitness. | 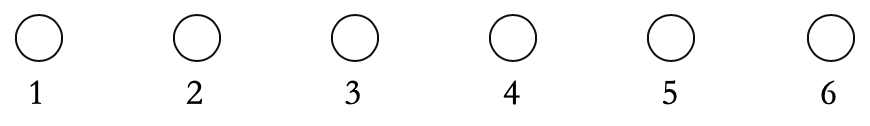 |

**Section 6: MaxDiff Motivational Effects**

Thank you for your patience in completing the previous questions. We would now like to invite you to play a small game involving a smartwatch. Please imagine the following scenario: It’s around 8 PM, you’ve had a tiring day of work or study and just want to relax and browse the web. Suddenly, your smartwatch pushes a notification. You will now see several different types of messages. Which message is most likely to motivate you to exercise? Which one is least likely? Please answer honestly.

1. Among the four messages below, which one is most likely to motivate you to exercise? Which one is least likely? (Relatively)
   (12 questions in total, according to the MaxDiff experimental design)

**Section 7: Hexad Gamification User Types and Dimensions of Gamification Drive**

Next, please rate your agreement with the following statements based on your real experience and preferences when using gamified applications (e.g., Ant Forest).
(Scale from 7: Strongly agree to 1: Strongly disagree)

| Statement | Rating |
| --- | --- |
| I like to strive for goals. | _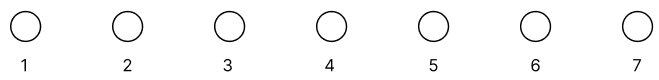_ |
| I enjoy emerging victorious out of difficult circumstances. | _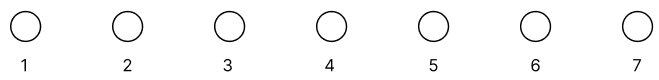_ |
| I like to immerse myself while playing games. | _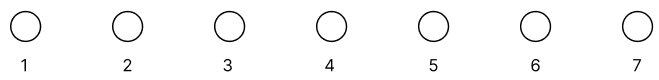_ |
| I dislike following rules. | _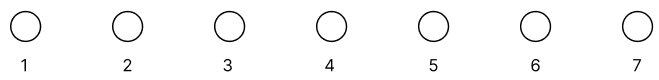_ |
| Interacting with others is important to me. | _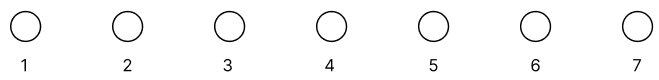_ |
| I often let my curiosity guide me. | _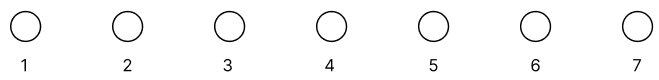_ |
| If the reward is sufficient I will put in the effort. | _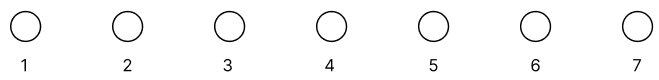_ |
| Rewards are a great way to motivate me. | _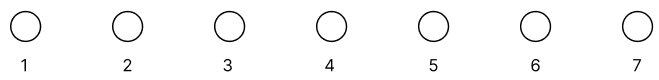_ |
| I like mastering difficult tasks. | _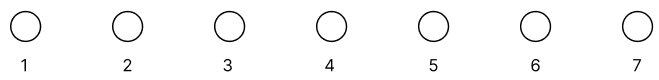_ |
| Return of investment is important to me. | _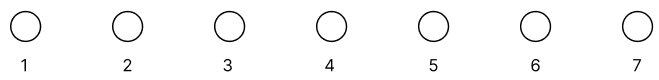_ |
| It is important to me to feel like I am part of a community. | _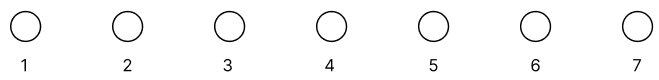_ |
| Opportunities for self-expression are important to me. | _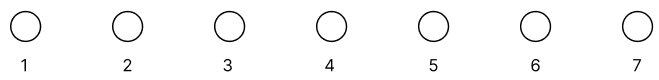_ |
| I like being part of a team. | _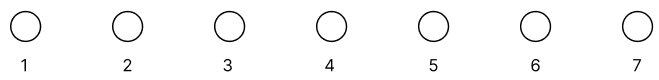_ |
| I long for recognition of others. | _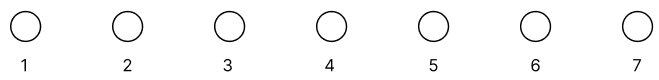_ |
| Storylines and world views of games are important to me. | _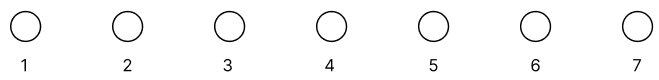_ |
| It makes me happy if I am able to help others. | _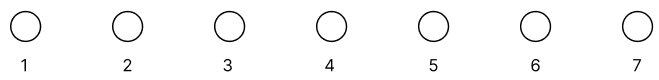_ |
| I want to see my progress every day. | _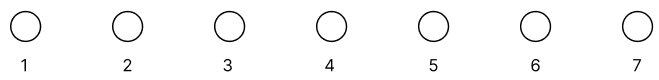_ |
| I see myself as a rebel. | _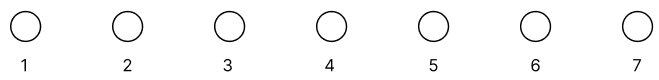_ |
| I like to overcome difficulties. | _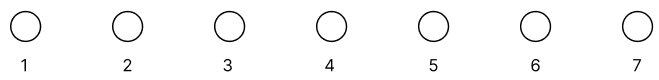_ |
| I enjoy group activities. | _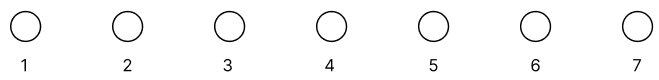_ |
| I like to share my achievements. | _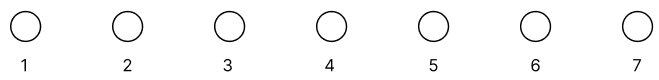_ |
| The wellbeing of others is important to me. | _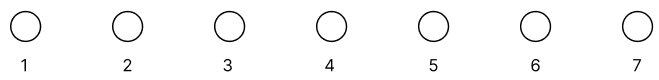_ |
| I like to provoke. | _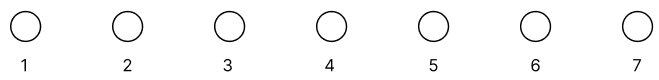_ |
| I want to become stronger. | _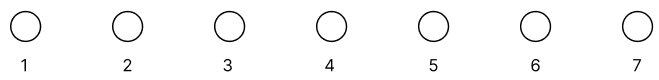_ |
| I like helping others to orient themselves in new situations. | _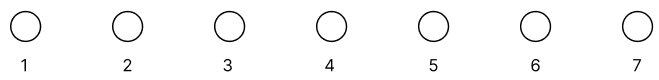_ |
| It is important to me to continuously improve my skills. | _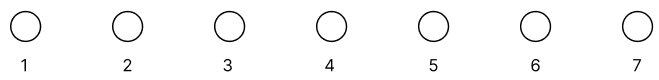_ |
| Being independent is important to me. | _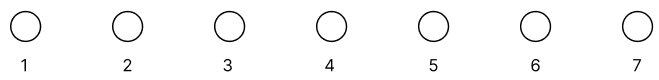_ |
| It is important to me to follow my own path. | _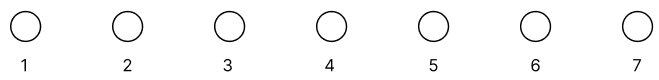_ |
| I like overcoming obstacles. | _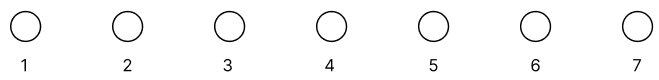_ |
| I like competitions where a prize can be won. | _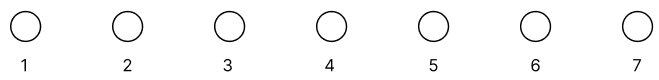_ |
| I like to question the status quo. | _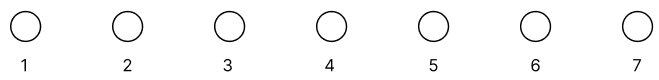_ |
| I like sharing my knowledge. | _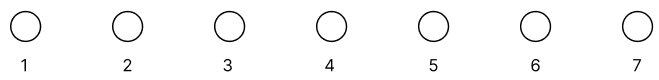_ |

**Section 8: Additional Questions**

1. The survey is almost over. Please recall how many days in the past 30 days you felt you had significant emotional problems (such as stress, anxiety, depression, etc.).
   (Number of days)
2. Your gender:
   A. Male
   B. Female
3. Your age:
   (Age in years)
4. Your occupation:
   A. Student
   B. Employed
   C. Other
5. How much do you like playing games?
   A. Don’t like playing games at all
   B. Generally like playing games
   C. Like playing games a lot
   D. Really enjoy playing games
6. How often do you play games?
   A. Rarely, only passively when the opportunity arises
   B. Occasionally, when I'm really bored
   C. Frequently, regularly once a week or every few days
   D. Very often, daily or almost daily

**Section 9: Conclusion**

You have completed all the questions in this survey. Thank you again for your valuable time!

The Original Questionnaire in Chinese

智能手表游戏化问卷

尊敬的先生/女士：

您好！我们正在进行一项关于“智能手表运动健康类应用程序游戏化设计”的研究，感谢您在百忙之中参与此次调查实验。本次实验采用匿名方式作答，您提供的宝贵意见和资料仅作学术研究之用，不会泄露您的任何信息。

本次实验可能需要15分钟左右，全程采用线上问卷的方式进行，由衷感谢您的支持与配合，认真完成答卷后我们会为您提供10元酬劳！

**第一节 条件筛选**

1. 您是否使用过智能手表/手环？

A.是 B.否（结束问卷并表示感谢）

**第二节 设备使用情况**

1. 您正在使用（或曾经使用）的智能手表/手环设备是：

A.Apple watch B.小米手表/Redmi Watch C.华为Watch D.Oppo Watch E.Samsung Galaxy Watch F.小米手环 G.华为手环 H.其他（）

2. 您使用智能手表/手环设备的时长是：

A. <3个月 B. 3-12个月 C. 1-2年 D. >=3年

3. 您使用智能手表/手环的频率是：

A.从不佩戴 B.只有运动时佩戴 C.偶尔佩戴 D.每天佩戴

4. 您使用智能手表/手环的主要目的是：

A.运动健身 B.健康监测 C.消息提醒 D.手机辅助 E. 其他（）

5.（多选）您使用智能手表/手环时常用的功能有哪些：

A.健身记录 B.消息通知 C.心率监测 D.步数记录 E.睡眠监测 F.血氧监测 G.卡路里监测 H.久坐提醒 I.经期记录 J.支付 K.提醒备忘 L.闹钟 M.天气 N.其他（）

**第三节 MaxDiff练习**

假如您的智能手表/手环上有一款运动健康类应用程序，以下我们将向您介绍它可能包含的一系列游戏化功能，以了解您的喜好。

本页介绍的四种游戏化功能中，哪种是您最喜欢的，哪种是您最不喜欢的？（相对来说）

（一共会展示四组这样的题目)

**第四节 偏好MaxDiff**

感谢您的耐心阅读，希望以上的介绍让您对各种游戏化功能有了初步的认识。

接下来我们想通过一个小实验来进一步了解您的喜好。以下共有十二道题，麻烦您诚实作答，非常感谢。

1.假如您正在使用一款智能手表上的运动健康类应用程序，下列游戏化功能中，哪种是您最喜欢的，哪种是您最不喜欢的？（相对来说）

（根据MaxDiff的实验设计方案一共有12道题）

13.（多选）假如您的智能手表/手环上有一款运动健康类应用程序，您希望它包含以下哪些游戏化功能？

A.社区 B.等级 C.目标 D.合作 E.虚拟货币 F.积分 G.竞赛 H.虚拟角色 I.挑战 J.排行榜 K.概览 L.反馈 M.共享 N.进度 O.徽章 P.叙事情节

**第五节 运动锻炼情况**

感谢您完成了以上实验，接下来我们想简要了解您目前的锻炼情况和智能手表/手环的使用情况。

1. 您目前运动的主要目标是：

A.保持健康 B.减肥减脂 C.增肌塑形 D.放松解压 E.兴趣爱好 F.其他（）

2. 您目前最常做的运动是：

A.散步 B.跑步 C.跳绳 D.游泳 E.登山 F.骑行 G.无氧健身类（器械、自重训练等） H.形体类（舞蹈、瑜伽、健身操等） I.多人球类（篮球、足球、排球等） J.双人球类（乒乓球、羽毛球、网球等） K.其他（）

3. 您目前的锻炼频率是：

A.一个月1次及以下 B.一个月2到3次 C.每周1到2次 D.每周3到5次E.大约每天1次

4. 您认为自己属于哪一类运动人群：

A.几乎不运动 B.普通运动人群（偶尔运动） C.运动爱好者（比较喜爱运动） D.运动狂热者

5.下面请您根据自己使用智能手表/手环的真实体验，标出您对下列题目的同意程度。

每个题目包含六个选项（1分表示非常不同意；2分表示不同意；3分表示比较不同意；4分表示比较同意；5分表示同意；6分表示非常同意）

| 描述 | 评价 |
| --- | --- |
| 我使用智能手表/手环是因为我想了解有关自身健康活动的更多信息。 | 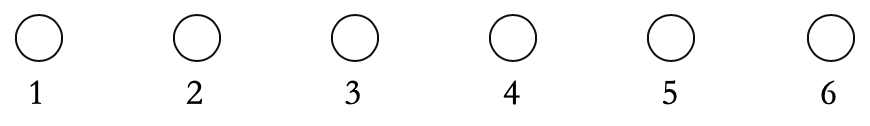 |
| 我使用智能手表/手环是因为达到我的步数或运动目标时它会鼓励我。 | 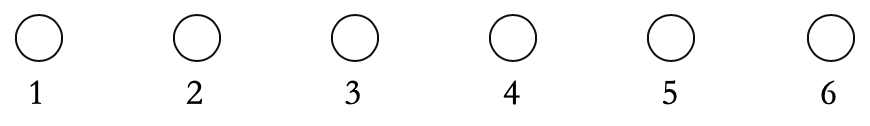 |
| 我使用智能手表/手环是因为我很享受监测并处理自己的健康活动数据。 | 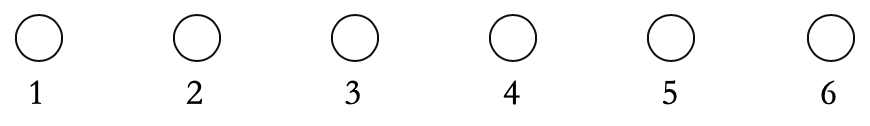 |
| 我使用智能手表/手环是因为监测并处理自己的健康活动数据很有趣。 | 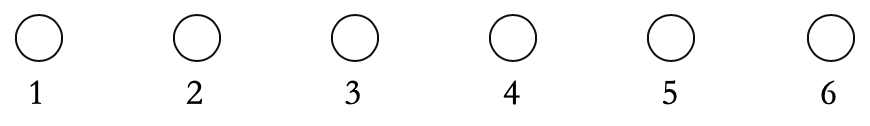 |
| 我使用智能手表/手环来避免自己运动太少。 | 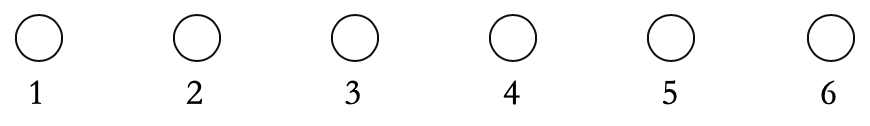 |
| 我使用智能手表/手环是因为它可以帮助我保持身体健康。 | 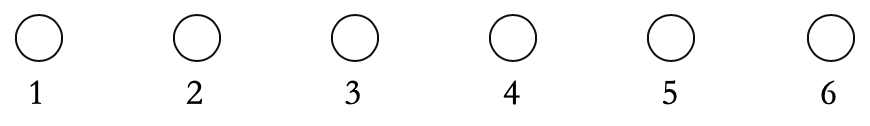 |

**第六节 激励Maxdiff**

感谢您的耐心填写。接下来我们想邀请您玩一个关于智能手表的小游戏，需要您假想以下场景：假设现在是晚上八点多，您今天已经辛苦地工作或者学习了一天，觉得有点累只想躺平上网，突然您手上的智能手表给您推送了一条消息。以下您将看到各种不同的消息推送，不知道哪条消息会对您触动最大呢？麻烦您诚实作答，非常感谢。

1.下列四条消息中，哪条最有可能激励你继续锻炼，完成今天所需的运动量？哪条最不可能？（相对来说）

（根据MaxDiff的实验设计方案一共有12道题）

**第七节 Hexad游戏化用户类型和游戏化驱动维度**

接下来，请您根据自己使用游戏化应用程序（如蚂蚁森林）时的真实感受和喜好，标出对下列题目的同意程度。每个题目包含七个选项（7分表示非常同意；6分表示同意；5分表示比较同意；4分表示一般；3分表示比较不同意；2分表示不同意；1 分表示非常不同意）

| 描述 | 评价 |
| --- | --- |
| 我喜欢努力达成目标。 | _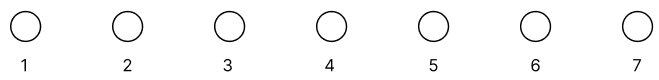_ |
| 从困境中获胜让我很享受。 | _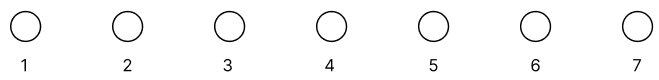_ |
| 玩游戏时，我想沉浸在游戏世界中。 | _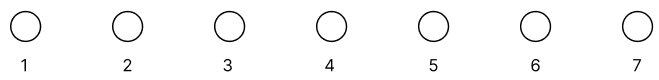_ |
| 我不喜欢遵守规则。 | _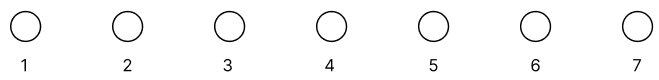_ |
| 与他人交流对我来说很重要。 | _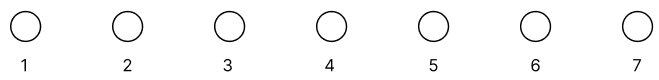_ |
| 我经常受好奇心驱使。 | _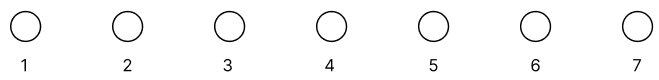_ |
| 如果有足够的奖励，我会努力。 | _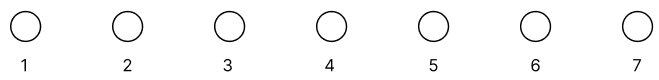_ |
| 奖励是激励我的好方法。 | _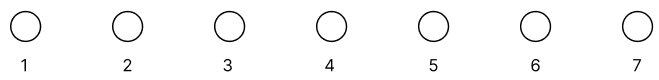_ |
| 我喜欢掌握困难的任务。 | _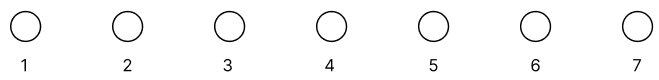_ |
| 投入有回报对我来说很重要。 | _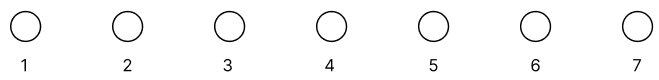_ |
| 感到自己是社区的一员对我来说很重要。 | _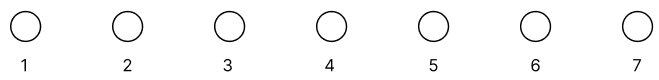_ |
| 表达自我的机会对我很重要。 | _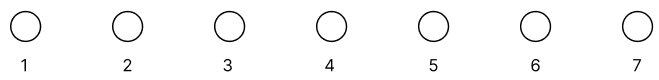_ |
| 我喜欢成为团队的一员。 | _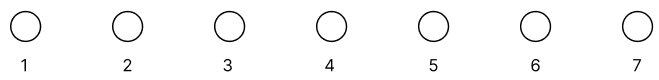_ |
| 我渴望得到他人的认可。 | _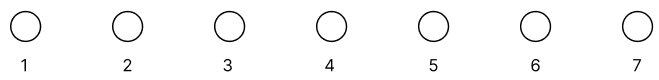_ |
| 我认为游戏的故事背景和世界观很重要。 | _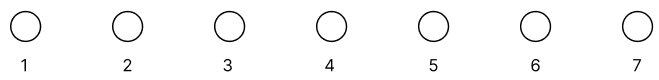_ |
| 如果我能帮助别人，我会很开心。 | _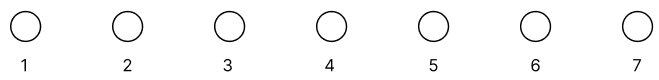_ |
| 我想看到自己每天的进步。 | _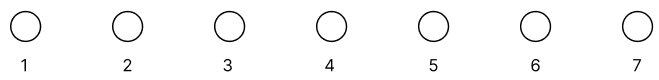_ |
| 我认为自己是一个反叛者。 | _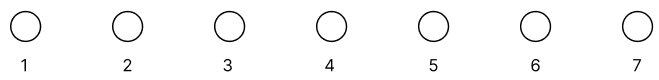_ |
| 我喜欢战胜困难。 | _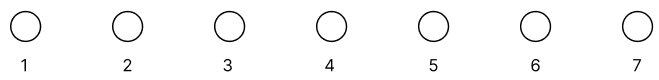_ |
| 我喜欢集体活动。 | _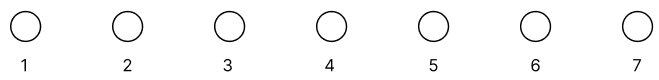_ |
| 我喜欢分享自己的成就。 | _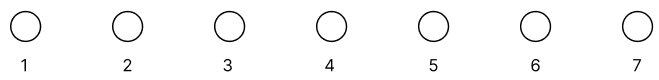_ |
| 别人的幸福对我很重要。 | _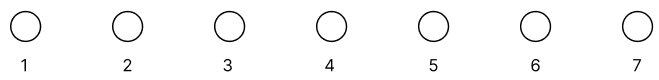_ |
| 我喜欢引发改变。 | _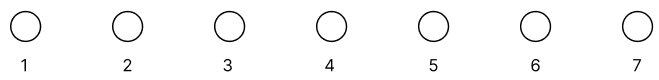_ |
| 我想变得更强大。 | _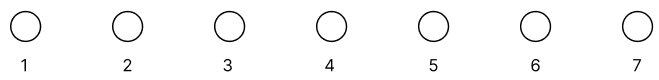_ |
| 我喜欢帮助别人适应新的环境。 | _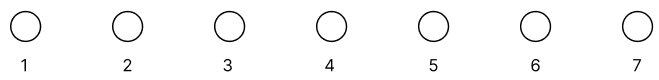_ |
| 对我来说，不断提高我的技能很重要。 | _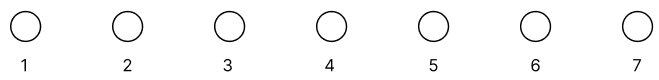_ |
| 保持独立对我很重要。 | _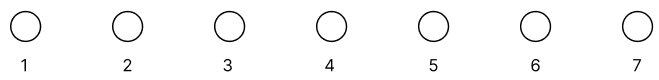_ |
| 对我来说，走自己的路很重要。 | _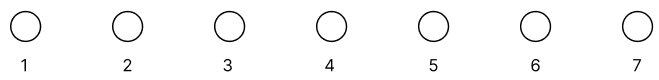_ |
| 我喜欢克服障碍。 | _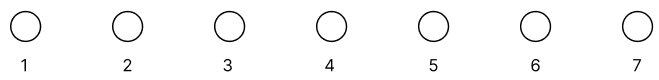_ |
| 我喜欢能得奖的比赛。 | _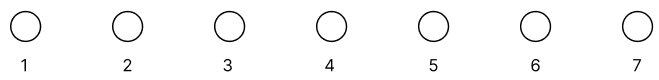_ |
| 我喜欢质疑现状。 | _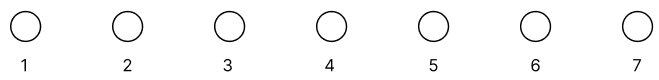_ |
| 我喜欢分享我的知识。 | _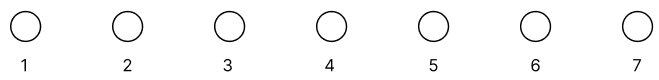_ |

**第八节 其他问题**

1. 本次调查即将结束，最后请您回想一下，在过去的30天里，大概有多少天您觉得自己有明显的情绪问题？（包括感到压力太大、焦虑、抑郁等）

（ ）天

2. 您的性别：

A.男 B.女

3. 您的年龄

（ ）岁

4. 您的职业：

A.学生 B.工作 C.其他

5. 您对游戏的喜爱程度属于：

A.完全不喜欢玩游戏 B.一般喜欢玩游戏 C.比较喜欢玩游戏 D.非常喜欢玩游戏

6. 您目前玩游戏的频率是：

A.极少，因缘际会，被动的接触 B.偶尔，实在无聊的时候玩 C.经常，定期的，每周或每隔一段时间 D.高，定期的，每天或每隔几天

**第九节 结束语**

您已完成本次调查的所有题目，再次感谢您的宝贵时间!
